# Orthoplastics Management of Open Lower Limb Fractures at a Major Trauma Centre: Audit of Adherence to BOAST4 Guidelines

**DOI:** 10.1016/j.jpra.2024.08.003

**Published:** 2024-08-20

**Authors:** Hester Lacey, Kaneka Bernard, Labib Syed, Evie O'Rourke, Yasmin Calvert-Ford, Joanna Bovis, Enis Guryel, Ian King

**Affiliations:** University Hospitals Sussex NHS Foundation Trust, Eastern Rd, Brighton and Hove, Brighton, BN2 5BE

**Keywords:** Orthoplastics, Open fractures, Major trauma

## Abstract

**Introduction:**

The British Orthopaedic Association Standards for Trauma and Orthopaedics (BOAST) guide the optimal management of open lower limb fractures. Adherence of the newly established Orthoplastic service at the Major Trauma Centre covering the Southeast of England was audited in relation to these standards.

**Materials and methods:**

Audit standards were produced. Data were collected using hospital records and the Trauma Audit and Research Network database. All open lower limb fractures managed between August 2020-August 2022 were included. Data collected included patient and injury demographics, and information related to initial and definitive management.

**Results:**

Overall, 133 patients were identified, 70 men and 63 women, with an average age of 58 years. Women had a higher average age (69 years) and ASA grade (71% ASA 3 or higher). Low-energy injuries occurred in 69% of women compared to 78% of high-energy injuries in men (p<0.001). Among them, 108 (81%) were debrided within 24 h. The average time to first debridement was 18.78 h, and 95 (75%) were definitively closed within 72 h, 76 with primary closure, 7 with split-thickness skin graft, 7 with local flap and 36 with free flap. Overall, the post-operative infection rate was 13% with 94% of these fractures definitively closed within 72 h.

**Conclusion:**

Most open lower limb fractures occurred in older women with higher ASA grade, from low-energy mechanisms. Most injuries were definitively managed as per the BOAST guidelines, but further efforts are required to improve the adherence to initial debridement targets, including training, appropriate resource allocation and implementation of procedures and proformas to guide injury management and improve documentation.

## Graphical abstract

**Figure:**
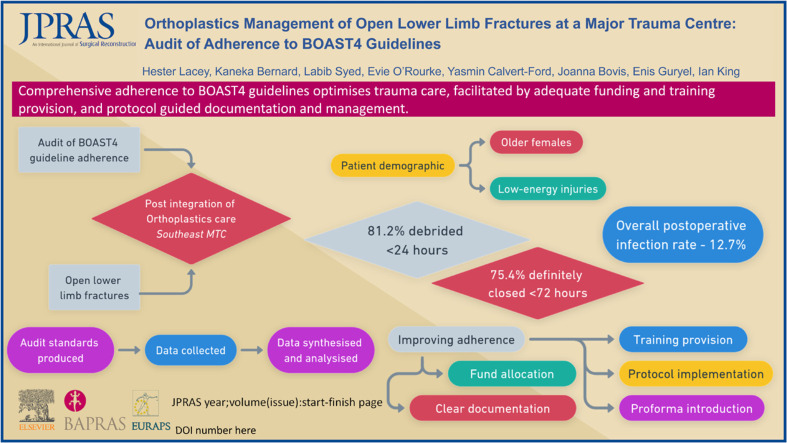

## Introduction

Before the establishment of major trauma networks, trauma care in the United Kingdom was fragmented and lacked standardised protocols.^1^ Patients with severe injuries, including open lower limb fractures, were managed at any acute hospital irrespective of the resource availability and clinical expertise in trauma management.^2^ This resulted in variations in care and access to specialist services, with implications on patient morbidity and mortality.^3^ The NHS Major Trauma Centres (MTCs) were developed in 2012 and strategically distributed to ensure adequate and timely access to specialist multidisciplinary trauma management from any location.^2^ This enabled the establishment of standards for timely and effective management of trauma patients.^4^

Open lower limb fractures are significant injuries which often result from high-energy trauma, with a high risk of morbidity and mortality.^5^ Timely and effective management is essential to reduce life- and limb-threatening complications and psychological consequences of these injuries, and the associated economic burden on the healthcare systems.^6^

The British Orthopaedic Association Standards for Trauma and Orthopaedics (BOAST) 4 guidelines provide UK evidence-based standards for the management of open lower limb fractures.^7^ These outline the optimal management of open lower limb fractures relating to prehospital care, early wound debridement, administration of prophylactic antibiotics, fracture stabilisation and appropriate soft tissue coverage to reduce the incidence of complications such as infection, limb loss and loss of life.^8^^,^^16^

The Trauma Audit and Research Network (TARN) was founded in 1990 to collect and analyse trauma data, support standardisation, improved consistency and quality of trauma care.^9^^,^^10^ The BOAST4 guidelines highlight the importance of submitting data to TARN, to monitor trauma network performance against national standards, facilitate auditing of centre outcomes and improve the consistency and quality of trauma care.^7^

Before the COVID-19 pandemic, a lack of on-site plastics presence at the Southeast MTC compromised BOAST4 guideline adherence. In August 2020, 5 plastic surgery consultants were introduced on-site, integrating Orthoplastic management of open fractures. This audit assessed the adherence to the BOAST4 guidelines for the management of open lower limb fractures following this change in structure. This includes defining our patient cohort, evaluating standard implementation and the influence of these changes on patient outcomes to identify areas of achievement, challenges and areas for further improvement.

## Methods

The BOAST4 guidelines were reviewed, and audit standards were produced and grouped into domains^7^ (Table 1). Data were collected retrospectively and included demographic information and data points relevant to each domain, including patient and injury demographics, and information relating to initial and definitive management. Patients suitable for inclusion were those with open lower limb fractures, defined as the long bones in the leg, hindfoot and midfoot. All ages and aetiologies of injury were included. Data were collected by review of hospital records, the TARN database and electronic operation records. Infection was confirmed by positive quantitative culture of fluid collection at the injury site. The data were then synthesised, analysed and compared to audit standards. Unpaired T-tests were used to calculate the significance for continuous data and chi-squared test was used for categorical data. The STROBE reporting guidelines were followed.

**Table 1:** Audit standards.

**Domain one: Prehospital and emergency department care**	
Realignment and splinting	Limb realignment and splinting prehospital
Neurovascular assessment	Assessment and documentation of neurovascular status
Prophylactic antibiotics	Intravenous prophylactic antibiotics within 1 h of injury
Imaging	Orthogonal views on radiograph for open fractures Trauma CT performed for polytrauma

**Domain two: Initial wound management**	

Time to wound excision	Within 12 h of injury for other solitary high-energy open fractures (likely Gustilo–Anderson Type IIIA and IIIB) and within 24 h of injury for all other low-energy open fractures
Vascular compromise	Immediate surgical exploration of devascularised limbs with revascularisation within 3–4 h
Compartment syndrome	Excision of necrotic tissue and closure of fasciotomy with STSG within 72 h
Photographs	Photographs taken before debridement and at other key stages of management
Antibiotics on induction of anaesthesia	Single dose of gentamicin 3 mg/kg on induction of anaesthesia
Saline wash	Low-pressure lavage with a high volume of tepid 0.9% saline

**Domain three: Definitive management**	

Skeletal stabilisation	Spanning external fixation if definitive stabilisation and immediate wound cover are not carried out at time of primary excision Definitive internal fixation carried out when immediately followed by definitive soft tissue cover Exchange from spanning external fixation to internal fixation within 3 days
Time to definitive soft tissue reconstruction	Performed at the same time as wound excision or within 72 hours of injury if it cannot be performed at the time of debridement
Amputation	Delayed primary amputation should be performed within 72 h

**Domain four: Patient outcomes**	

PROMS	Psychological difficulties should be recognised, and support given by trauma teams, with referral to trained mental health clinicians for evidence-based treatment where psychological difficulties are negatively impacting functioning and recovery
Rehabilitation	Patients requiring inpatient rehabilitation should be assessed within 10 days Recovery after severe open tibial fracture should be assessed 12 months after injury using the EuroQol-Five Dimensions (EQ-5D) tool

## Results

Overall, 133 patients who met the inclusion criteria were identified between August 2020-August 2022 (Table 2). Gustilo and Anderson Classification (G&A) scores were used to classify the injuries and were calculated perioperatively. The Gustilo and Anderson Classification (G&A) score is a scoring system used to determine fracture severity by considering the degree of energy of injury, extent of soft tissue injury and level of contamination.

**Table 2:** Baseline characteristics of the study sample.

Baseline characteristics								
	Gender		Age (years)					
	n	%						
**Overall**	133	100	58					
**Male**	70	53	48.2					
**Female**	63	47	69.1					

Mechanism	Fall <2 m		Fall >2 m		Motor vehicle collision			
	n	%	n	%	n	%		
**Total**	73	55	14	11	46	35		
**Male**	23	32	12	86	35	76		
**Female**	50	68	2	14	11	24		
**G&A**	**I**		**II**		**IIIa**		**IIIb**	**IIIc**
**Total**	2	2%	39	29%	27	**Total**	2	2%

Legend: G&A - Gustilo–Anderson Classification.

**Figure 1:**
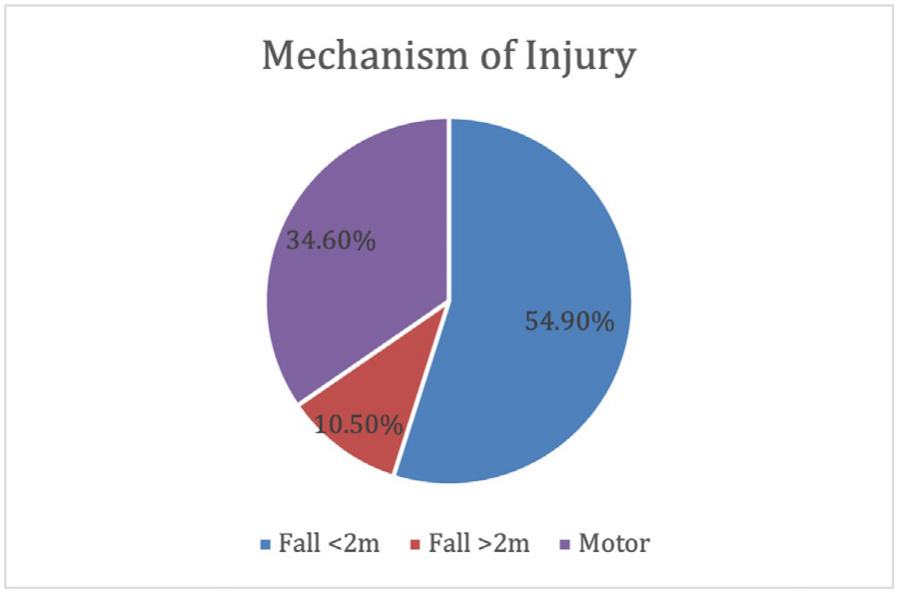
Mechanisms of injury.

Injuries were classified by mechanism as under 2 m, over 2 m or involving motor vehicles (Figure 1).

G&A score was compared to mechanism of injury (Table 3). There was a statistically significant association between mechanism and G&A score (p<0.001).

**Table 3:** Association between G&A and mechanism of injury.

G&A		1		2		3a		3b		3c		p
		n	%	n	%	n	%	n	%	n	%	
Mechanism	< 2 m	1	50	34	87	17	63	21	33	0	0	<0.001
	>2 m	0	0	1	3	3	11	10	16	0	0	
	Motor vehicle	1	50	4	10	7	26	33	52	1	100	

## Domain one: Prehospital and Emergency Department Care

The data were analysed in relation to the audit domains and BOAST4 guidelines. Totals under 133 indicate where documentation was unclear or unavailable (Table 4). Data relating to this domain were limited by incomplete or unavailable documentation, highlighting the limitations of paper documentation particularly with regional interhospital transfers.

**Table 4:** Prehospital and emergency department care.

	Realignament¹		NV Assessment²		Antibitiocs³		Imaging⁴	
	n	%	n	%	n	%	n	%
**Yes**	45	51	120	94	87	76	133	100
**No**	44	49	8	6	28	24	0	0
**Total**	89		128		115		133	

Legend: 1 - Limb realignment and splinting prehospital, 2 - Assessment and documentation of neurovascular status, 3 - Intravenous prophylactic antibiotics within 1 h of injury, 4 - Orthogonal views on radiograph or trauma CT.

## Domain two: Initial wound management

The average time to first debridement was 18.77 h. Mechanism of injury was used to classify injuries as high-energy (involving a fall of >2 m or motor vehicles) or low energy (involving a fall of <2 m) (Table 5).

**Table 5:** Initial wound management.

	n	Debridement	n	%
High-energy	60	<12 h	15	25
		<24 h	51	85
Low energy	73	<24 h	56	77
All injuries	133	<24 h	108	81

Legend: High-energy - Gustilo–Anderson Type IIIA and IIIB injuries.

In this study, 25 patients were debrided over 24 h. Among them, 10 represented transfers from other hospitals, increasing the time until operative Orthoplastics management. Four patients died before operative management. Seven patients were debrided between 24.5 and 29.7 h after injury. One patient was debrided at 31.01, one at 38.7, one at 41.18 and one at 51.1 h, all of which represented falls in older adults with a long lie before ambulance transfer and management at the MTC. This suggests that in most cases, debridement within 24 h was achieved, with the exceptions relating to clinical circumstances or interhospital transfer.

One patient had vascular compromise and had a below-knee amputation. The time to first debridement in this case was 15.21 h, with 19.6 h until definitive soft tissue coverage.

Five patients had compartment syndrome, 100% of which were definitively closed within 72 h, with an average of 22.5 h until definitive soft tissue coverage.

### Initial theatre management

**Figure 2:**
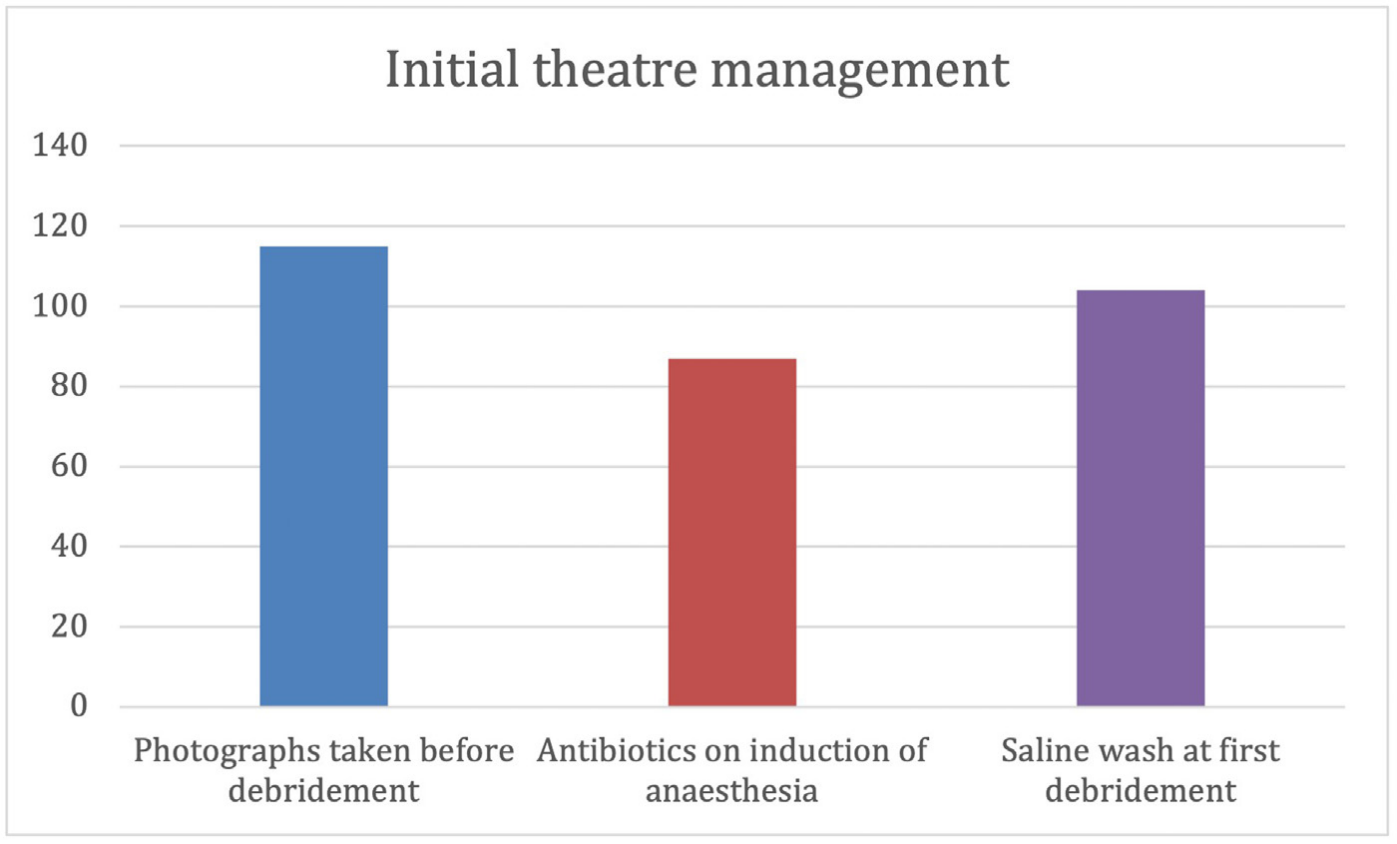
Initial theatre management.

Photographs of wounds were available for 115 patients (86%). The 18 injuries without photographs represented immediate transfer to the theatre on arrival. Antibiotics on induction of anaesthesia were documented for 87 patients (65%). Saline wash performed at initial debridement was documented in 104 (78%) operation notes (Figure 2).

This represents a challenge of paper documentation, where illegibility or lost documentation influences how representative the data collected is of the true management, and a learning point for teams to reflect on the importance of documentation for subsequent evaluation of practice.

## Domain three: Definitive management

Overall, 126 patients had definitive management at the MTC. Four patients died and 3 were transferred to other hospitals.

### Skeletal stabilisation

**Figure 3:**
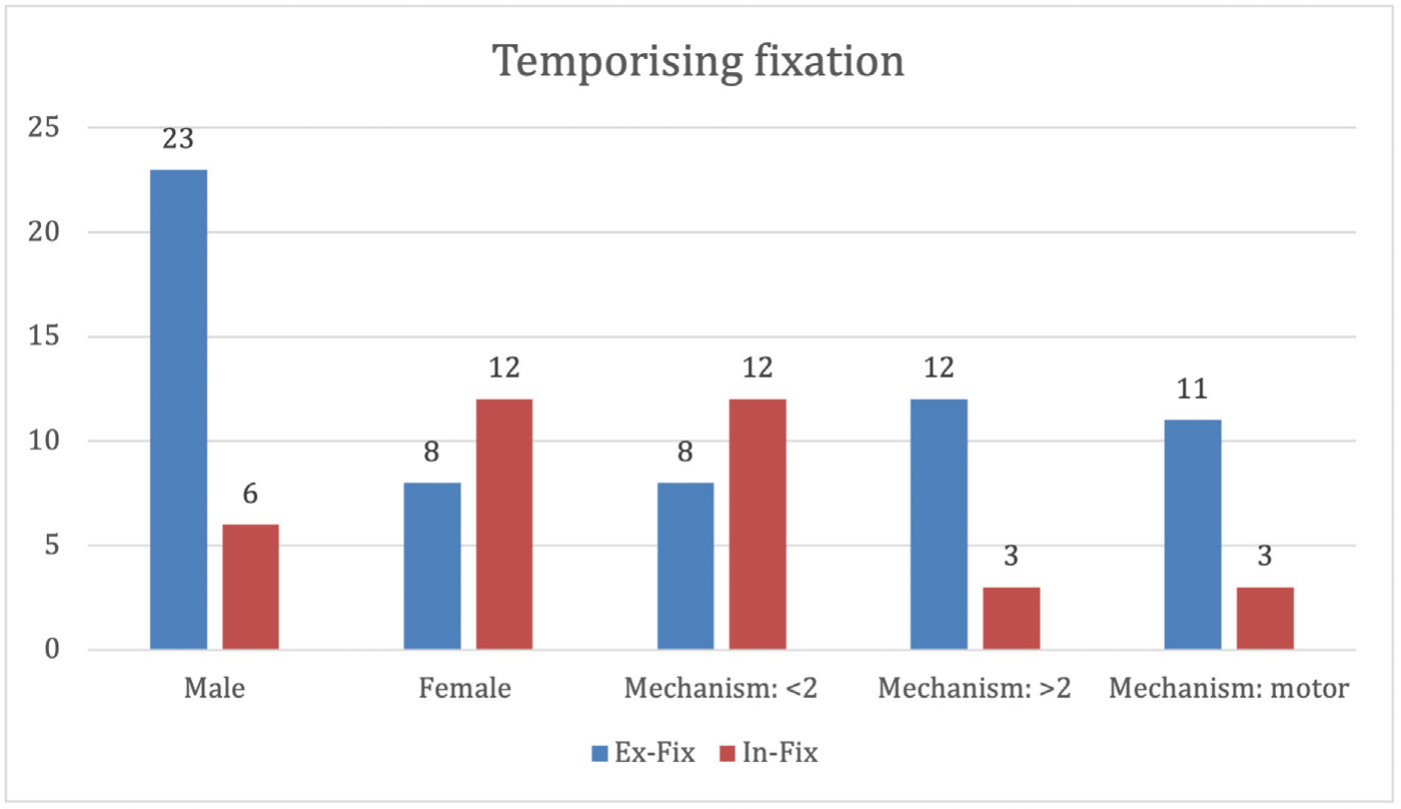
Demographics of patients receiving temporising fixation.

Seventy-seven patients were managed with definitive internal fixation following debridement at the first procedure. Forty-nine were managed with temporising fixation, 31 by external fixator (Ex-Fix) and 18 by internal fixator (In-Fix). Features of patients receiving temporising fixation are included in Figure 3.

In men, the mechanisms of injury involving falls >2 m and those involving motor vehicles were significantly more likely to be managed with Ex-Fix than injuries in women or those involving mechanisms of injury <2 m (p<0.001).

### Definitive internal fixation

Time to definitive internal fixation within 72 h is described in Table 6. The average time to definitive internal fixation was 77.4 h.

**Table 6:** Definitive internal fixation.

	n	%	p
<24 h	56	44	
<72 h (cumulative)	95	75	<0.001
>72 h	31	26	

Among the 31 patients closed over 72 h, 3 were related to transfers from other hospitals, influencing time to definitive management. The time to definitive internal fixation in this population ranged from 75.2-1058.1 h. Fourteen of these patients were closed at <120 h. Closures beyond this time were related to complex polytrauma with several patients being managed in ITU, and 4 patients with infections complicating staged operative management. Moreover, 109 (87%) had definitive closure within 7 days.

**Figure 4:**
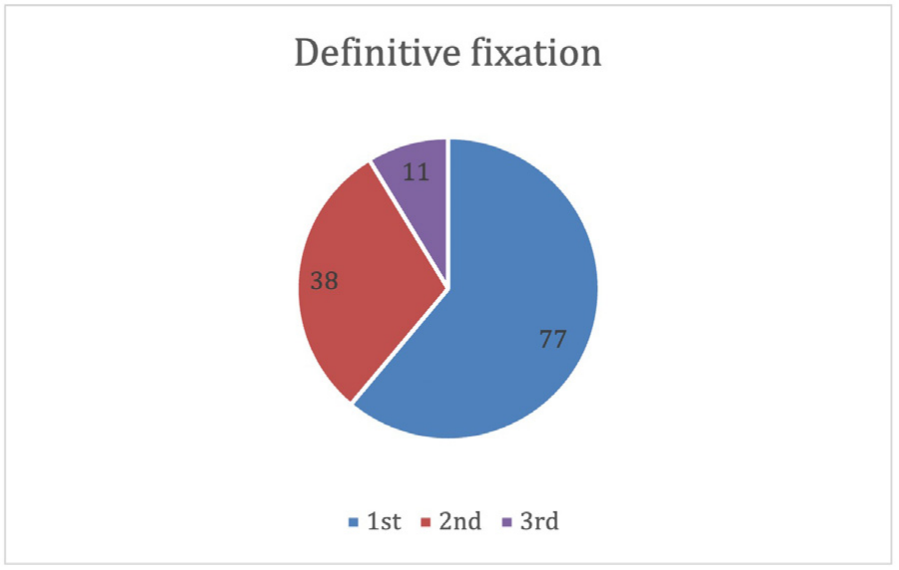
Timing of definitive fixation.

Definitive internal fixation was performed for 77 patients at the first operation, 38 at the second operation and 11 at the third operation (Figure 4).

Four patients had >3 operations, including 2 secondary amputations related to infection, and 1 related to flap failure.

The average time of exchange from temporising to definitive fixation was 126.7 h.

### Soft tissue reconstruction

**Figure 5:**
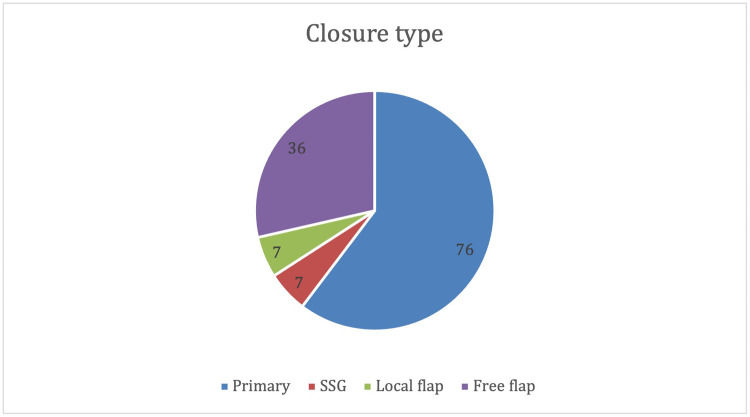
Closure type.

Seventy-six (60%) injuries were managed with primary closure, 7 with split-thickness skin graft (STSG), 7 with local pedicle flap and 36 with free flap (Figure 5).

In patients with compartment syndrome, 4 required free flap reconstruction, with 1 being managed with primary closure.

### Amputations

**Figure 6:**
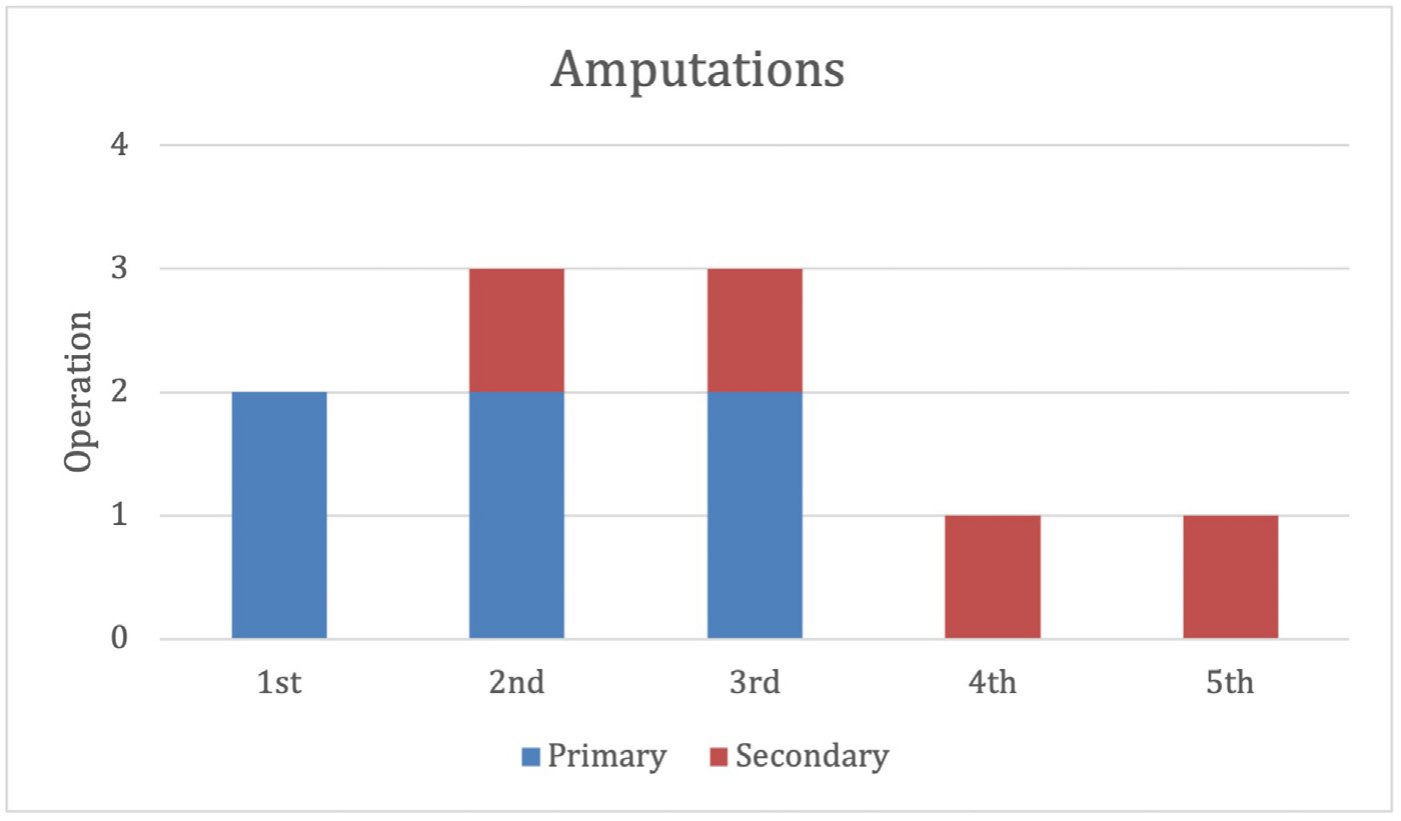
Primary and secondary amputations.

Ten patients had amputations (Figure 6). Six were primary amputations, with 2 being performed immediately after debridement. Four secondary amputations were performed between 13-44 days after the initial procedure, all related to soft tissue infection.

## Domain four: Patient outcomes

**Figure 7:**
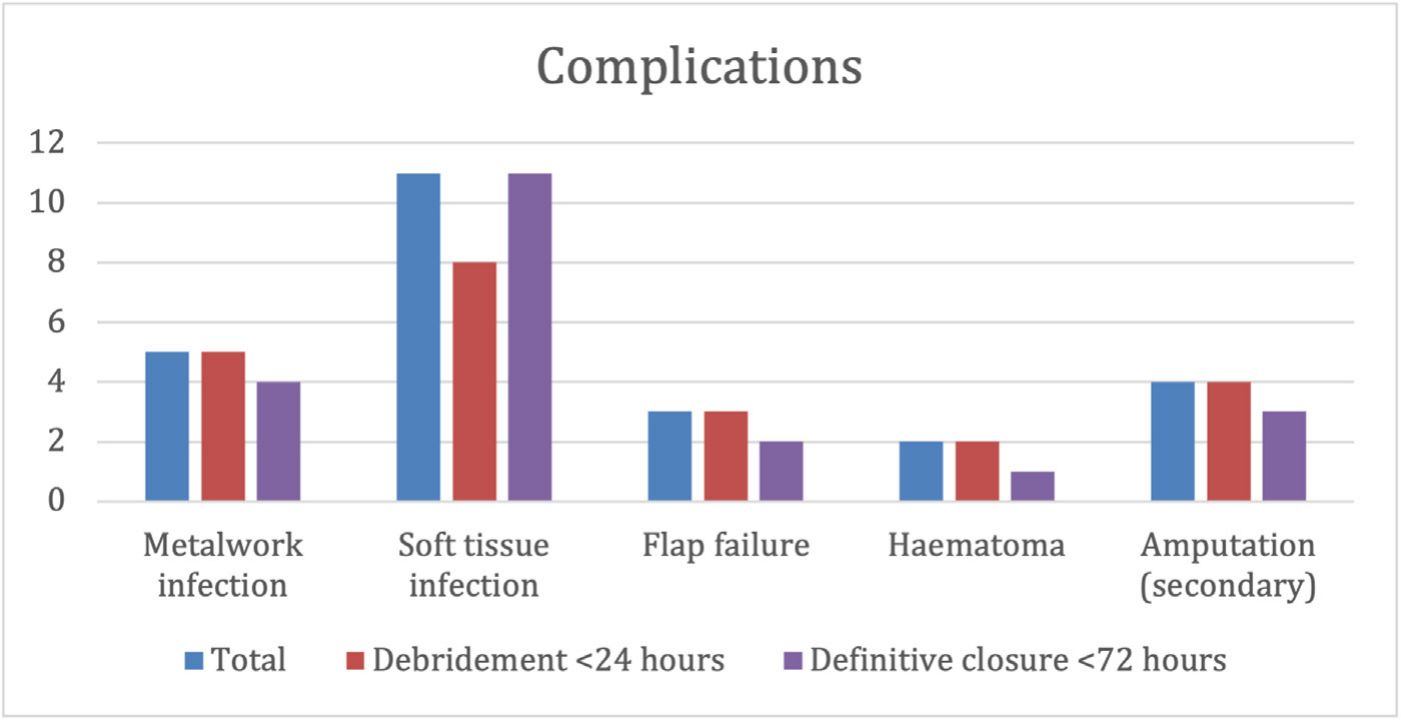
Complications.

**Figure 8:**
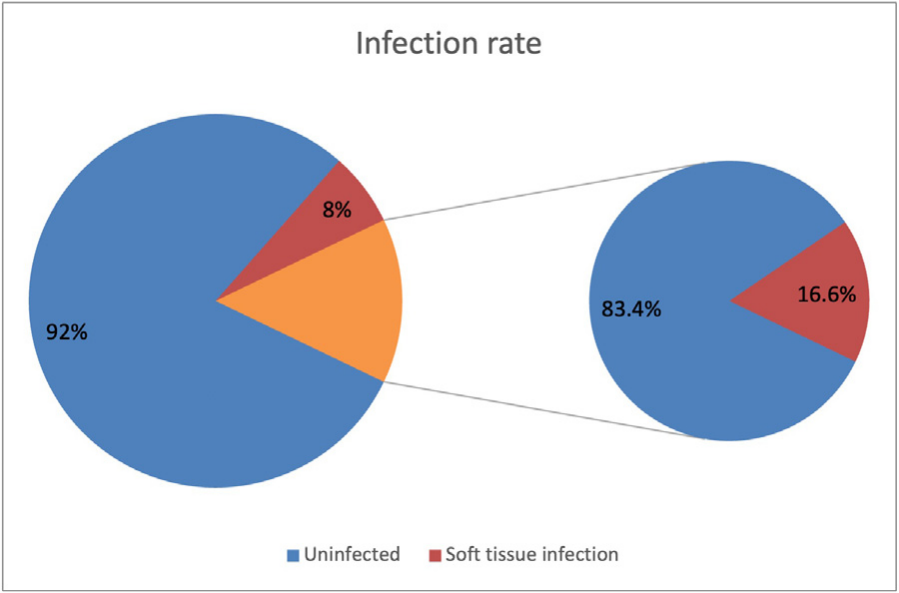
Infection rate related to the time to initial debridement.

Sixteen patients developed post-operative infection, 5 metalwork and 11 soft tissue infections. Thirteen patients with infection were debrided under 24 h, and 15 definitively closed within 72 h (Figure 7). There was an 8% soft tissue infection rate in those debrided within <24 h, compared to 17% in those debrided >24 h later, suggesting that early debridement reduced the incidence of soft tissue infection (Figure 8).

**Figure 9:**
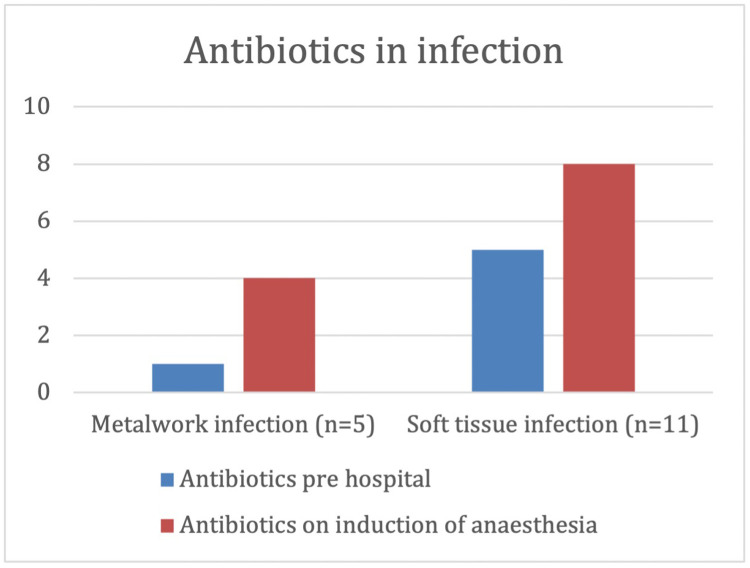
Antibiotic use in patients who developed infections.

In this study, 80% and 73% of patients who developed metalwork and soft tissue infection had antibiotics on induction of anaesthesia and 20% and 36% received prehospital antibiotics. Moreover, 74% of patients without infection received prehospital antibiotics (Figure 9). Prehospital antibiotics within 1 h of injury was associated with a significantly reduced risk of post-operative infection (p<0.001).

The average length of stay was 24 days. All patients received physiotherapy post-operatively.

## Discussion

This is the first audit of the management of open lower limb fractures according to the BOAST guidelines at the Southeast MTC since the integration of Orthoplastics care. Collaboration between orthopaedic and plastic surgeons is crucial for comprehensive and guideline-adherent management of these injuries, ensuring a multidisciplinary approach to complex injuries, and improving the overall patient outcomes and rates of limb salvage.^8^

### Demographics and mechanism of injury

The BOAST4 guidelines characterise low-energy injuries as those resulting from <2-metre fall or distance to impact and exclude motor vehicle accidents.^9^ Our results demonstrated that 55% of the open fractures managed at this MTC occurred in older women following low-energy trauma, with more comorbidities, suggested by the higher average ASA grades (Supplement one). Older adults, particularly those with pre-existing systemic disease and comorbidities, have an increased risk of falls due to environmental, medical and iatrogenic factors such as immobility, impaired balance and coordination, polypharmacy and sarcopenia. Moreover, this population are more vulnerable to serious injury after minor trauma, owing to reduced bone mineral density because of age, osteoporosis, steroid use, systemic disease and declining oestrogen levels during post menopause in women, increasing fracture risk.^10^ Systematic identification and addressal of individual patient factors that may negatively influence perioperative outcomes is required, to ensure a comprehensive, patient-centred approach to trauma management, aiding effective post-injury rehabilitation and reducing the risk of further injuries.

### Time to initial debridement

The Gustilo–Anderson Classification (G&A) score is a scoring system used to determine fracture severity, considering the energy of mechanism, extent of soft tissue injury and injury contamination.^11^ Progression from type 1 to type 3 suggests more extensive soft tissue and bone damage, and a higher risk of neurovascular and limb compromise and complications.

The BOAST4 guidelines recommend that the initial debridement should occur within 12 h for high-energy injuries (likely G&A 3a and 3b) and 24 h for all other injuries. Early debridement with irrigation of the contaminated wounds and resection of necrotic or vascularised tissue is essential in reducing bacterial load in open wounds, reducing the risk of acute and chronic infection and complications, including fracture non-union, flap failure, need for further operative management and systemic morbidity, and mortality.^12^ High-energy injuries and G&A scores >3 are associated with more complex injuries, likely requiring more extensive and prolonged operative management and an increased complication risk.^13^ Early debridement optimises the surrounding soft tissues and increases the likelihood of bony union, successful soft tissue coverage and restoration of functional status.^14^

G&A scores were incompletely documented in the notes reviewed; hence, several were calculated retrospectively. Most injuries were type 3 or above, with type 3 injuries typically related to mechanisms of injury involving a fall >2-metres or motor vehicles. Although G&A score can be estimated on the initial injury evaluation, the full extent of injury may not be evident until surgical debridement, hence establishment of scores G&A3 or above may not be possible before the patient enters the theatre, and in this case, often was not definitively evaluated and documented.^11^ The BOAST4 guidelines offer no further definition of high- versus low-energy trauma. Adherence of this centre to the BOAST4 guidelines for debridement of high-energy injuries <12 h was negatively influenced by a lack of systematic early classification of injury G&A score and need for early operative management.

Our audit highlighted that 81% of patients managed at this MTC were debrided within 24 h, comparable with outcomes from other MTCs (90% stabilisation within 24 h^15^). Given the limitations in data collection and confirming the high-energy status, this suggests that most injuries were debrided within an appropriate and effective timeframe within the BOAST4 guidelines. Improving the overall adherence to time to initial debridement guidelines requires implementation of clear guidance for calculating G&A score in the emergency department, which could include modification of trauma clerking proformas and clinician training in open fracture assessment.

### Definitive Management

The BOAST4 guidelines highlight the use of temporary fixators for fracture stabilisation if definitive closure is not possible at the first debridement. Typically, external fixation is required for injuries with significant bone and soft tissue loss and limb deformity, commonly polytrauma injuries, to stabilise injuries while optimal timing for definitive fixation and closure is determined.^15^ In this centre, temporising fixation was commonly used in high-energy mechanisms of injury and male demographics, in line with the common indications for temporising fixation.

The BOAST4 guidelines outline that definitive soft tissue reconstruction should be performed within 72 h if not performed at the time of initial debridement. Compliance with definitive closure within 72 h was at 75%. This is comparable or better than the 72-hour outcomes of other MTCs (72%^8^ and 21%^16^ compliance with 72-hour targets, respectively). The overall average time to definitive internal fixation was 77.4 h. This suggests that with the identification and implementation of steps to streamline management, such as timely interhospital transfer, early Orthoplastics consult and expedited time to theatre, compliance with definitive closure within 72 h may easily be brought in line with the BOAST4 guidelines.

### Missing data

Prehospital and Emergency Department care (Domain one) and initial wound management (Domain two) represent the areas with the most missing data, including clear documentation of limb realignment and splinting, neurovascular assessment, use of prehospital and intraoperative antibiotics and intraoperative saline wash. This highlights the limitation of paper documentation particularly with interhospital transfer, where clinical notes were not available for data collection, or documentation was missing or incomplete. To improve this, the introduction of a clear, accessible referral proforma when accepting patients for transfer in keeping with the BOAST4 initial management guidelines would assist with ensuring intravenous antibiotic administration, limb splinting and neurovascular assessment are provided and documented prehospital. To improve intraoperative documentation, a proforma for operative notes is recommended, to guide clear documentation on the use of intraoperative antibiotics and saline wash.

### Infection rate

Infection risk following open lower limb fracture is linked to the extent of tissue damage, wound contamination and damage to surrounding vasculature.^17^ Compliance with the BOAST4 guidelines is associated with reduced risk of acute and chronic infection, including antibiotic administration prehospital and during initial debridement, and early wound debridement.^6^ Antibiotic administration prehospital within 1 h of injury had a significant relationship with development of infection in our cohort, suggesting improving prehospital adherence to the BOAST4 guidelines offers a significant opportunity for reducing infection rates and perioperative complications including secondary amputations.^6^

### Patient outcomes

Patient-reported outcome measures (PROMs) are important tools for assessing patient perspectives on health, quality of life and the effects of medical interventions.^18^ There are several challenges associated with obtaining PROMs. Collecting and managing PROM data is resource-intensive, requiring appropriate systems and expertise for data entry, storage and analysis. Equally, patient compliance, recall and response bias can influence data collection and reliability. The data at this MTC are lacking in clinical records and TARN data. To improve compliance with the BOAST4 guidelines relating to patient outcomes, a dedicated tool for post-operative psychological assessment may be beneficial, facilitating the identification of psychological difficulties that may influence perioperative morbidity and recovery and prompt early referral to mental health services. Improving compliance with 12-month assessment using the EuroQol-Five Dimensions (EQ-5D) tool is also highlighted; however, 12-month follow-up in this cohort was impacted by the COVID-19 pandemic, limiting the ability of outpatient clinics to comprehensively adhere to these targets.

## Conclusion

The Orthoplastics team at the UK Southeast MTC has made significant strides in adhering to the BOAST4 guidelines since 2020, emphasising the importance of multidisciplinary collaboration and standardisation of trauma care. This is the first audit of adherence to the BOAST4 guidelines since the integration of Orthoplastics care. These results are invaluable to stimulate discussion and strategies to further improve the management of open lower limb fractures in this unique patient demographic, including optimal allocation of funds, training, intrahospital proformas and protocols to improve guideline compliance. Key limitations of this audit included incomplete and inconsistent documentation influencing the completeness of data collection. These experiences and challenges offer valuable lessons and contribute to the broader national effort to enhance the quality of trauma care.

## Conflict of interest

The authors have no conflicts of interest to declare.

## Appendix Supplementary materials

Image, application 1
