# Supplementary material for: Orthoplastics Management of Open Lower Limb Fractures at a Major Trauma Centre: Audit of Adherence to BOAST4 Guidelines
Source: JPRAS Open. 2024 Aug 20;42:133–45. doi: 10.1016/j.jpra.2024.08.003 (PMC11415631; doi:10.1016/j.jpra.2024.08.003)
Supplement: Supplementary file 1 [file mmc1.docx]

**Supplement one**

| **ASA grade** | **1** | | **2** | | **3** | | **4** | |
| --- | --- | --- | --- | --- | --- | --- | --- | --- |
| **Total** | 34 | 37% | 33 | 36% | 18 | 20% | 6 | 7% |
| **Male** | 21 | 62% | 21 | 64% | 6 | 33% | 1 | 17% |
| **Female** | 13 | 38% | 12 | 36% | 12 | 67% | 5 | 83% |

*Legend: American Society of Anesthesiologists (ASA) physical status classification provides a categorisation of a physiological status that can help predict operative risk.*
